# BSTS synthesis guided by CALPHAD approach for phase equilibria and process optimization

**DOI:** 10.1038/s41598-023-30976-3

**Published:** 2023-03-09

**Authors:** Husain F. Alnaser, Taylor D. Sparks

**Affiliations:** 1 Department of Material Science and Engineering, University of Utah, Salt Lake City, UT 84112 USA; 2 Chemistry Department, University of Liverpool, Liverpool, United Kingdom

**Keywords:** Materials science, Electronic devices

## Abstract

This work presents a new method for processing single-crystal semiconductors designed by a computational method to lower the process temperature. This research study is based on a CALPHAD approach (ThermoCalc) to theoretically design processing parameters by utilizing theoretical phase diagrams. The targeted material composition consists of Bi–Se_2_–Te–Sb (BSTS). The semiconductor alloy contains three phases, hexagonal, rhombohedral-1, and rhombohedral-2 crystal structures, that are presented in the phase field of the theoretical pseudo-binary phase diagram. The semiconductor is also evaluated by applying Hume–Rothery rules along with the CALPHAD approach. Thermodynamic modelling suggests that single-crystals of BSTS can be grown at significantly lower temperatures and this is experimentally validated by low-temperature growth of single crystalline samples followed by exfoliation, compositional analysis, and diffraction.

## Introduction

Using topological insulating materials for quantum computing is a promising emerging technology that will have vast implications on industry and society as a whole^1^. For decades, computers have been developed to be smaller in size, faster in operation, and larger in memory size^2^. However, as transistors reach the nanometer scale, low dimensional phenomena such as tunneling become more problematic, and radical alternative approaches, such as quantum computing, must be considered. Quantum-matter heterostructures have emerged as a versatile approach for controlling quantum states in materials^2^.

Advanced semiconductor devices use heterostructure as a building block because of the precise control over the states and motions of charge carriers. The heterostructure advantages stem from harvesting a unique design where a thin layer of material grows on a different thin layer of material that has different bandgaps and lattice constants providing an easy tunneling effect for the carriers to promote quantum wells^3^. The heterostructure can be synthesized by an epitaxial growth process such as molecular beam epitaxy, liquid phase epitaxy, and chemical vapor deposition. In our previous studies, BiSbTeSe_2_ (BSTS) alloy was proven to be an excellent candidate for a 3D topological insulator utilizing the heterostructure approach because each layer provides a unique set of properties that when combined promote the quantum hall effect^4–6^. The theoretical predictions suggest that the interior of a topological insulator material sample works like an insulator, while the exterior, namely, the metallic surface, has a Dirac cone dispersion and a helical spin structure^7^. Despite the availability of other compounds that serve as a good topological insulator, BSTS was favored because it provides a stable platform for characterizing without disturbing the bulk electron states^8^.

Quantum computers need special topological insulator materials to optimize their capacity^9^. BSTS single crystals have drawn researchers’ attention because of the Dirac quantum hall effect^10^. Consequently, BSTS single crystals have been studied by many researchers because they offer unmatched opportunities for creating new novel quantum state possibilities^11^. The manufacture of quantum-matter heterostructures relies on the exfoliation of thin sections of high-quality materials, such as single crystals^12^. Many researchers have attempted to make BSTS single crystals through trial-and-error high-temperature synthesis. To date, there are only limited reports of process design principles despite the requirement for improved purity, control of composition, larger crystals, and reduced energy consumption upon synthesis.

In other fields such as solid-solution strengthening, thermodynamic modeling is used regularly to guide synthesis and provide process design principles^13^. For example, the simple Hume–Rothery rules provide a starting point for alloy design via solid-solution strengthening. The four rules are: If the solute is more than 15% different in size from the solvent, the solubility of the species is limited. If the solute has a large difference in electronegativity, then the solvent forms intermetallic compounds and limits the alloy solubility. If the solute and the solvent have the same valence, complete solubility occurs; otherwise, solubility would be limited. Lastly, if the solute and solvent have different crystal structures, then solubility would be limited. These rules work best for binary alloys but can be extended to even more complex systems if these are treated as a pseudo-binary alloy where the composition is held constant (the solvent) except for the addition of one constituent (the dopant). The generation of pseudo-binary diagrams is non-trivial and relies on the aid of thermodynamic modeling such as CALPHAD (Computer Coupling of Phase Diagrams and Thermochemistry) ThermoCalc software. CALPHAD allows researchers to compute thermochemical properties and phase diagrams^14^. These computations are precise and accurate to a high degree. A study performed by Fetzer comparing the phase diagrams of experimental data versus the CALPHAD approach to determine the precession of thermodynamic properties and phase fields for Mg alloys. He concluded that the CALPHAD approach precisely predicted his experimental data^15^.

Polycrystalline materials have limits and cannot perform as required because of the formation of grain boundaries that reduce electron mobility because of electrostatic potentials and lattice mismatch^16^. Grain boundaries are also susceptible to enhanced corrosion because of their higher energy state. Single crystals, on the other hand, have excellent corrosion resistance and enhanced electronic mobility that have made these materials staples for modern semiconductor industries^17^. Topological insulator single crystals have recently been employed in heterostructure architectures because of enhanced electron transport, control of specific crystal orientation, and interfaces^18^. The synthetic techniques used for these topological insulating single crystals are quite varied and span zone melting, Czochralski method, Bridgman method, saturated solution growth, flux method, and others^19^.

The literature review shows that a common temperature for single-crystal growth of BSTS is in excess of 850 °C without thermodynamic justification^20,21^. BSTS is composed of several toxic elements with dangerous health implications for those exposed^22–25^. Consequently, relying on a high-temperature synthesis route has potential disadvantages because of the ease of contamination that is possible because of the excessive volatilization of low-melting-point constituents. In addition, most single-crystal growers use quartz ampoules to isolate the BSTS materials from oxidation during crystal growth, and tube rupture is a regular concern. A tube-rupture formula can determine the ampoule thickness needed to withstand the internal pressure generated because of interior component volatilization^26^. Note that the formula cannot be used when the internal pressure exceeds 100 psi = 6.89 bar.

**Figure 1:**
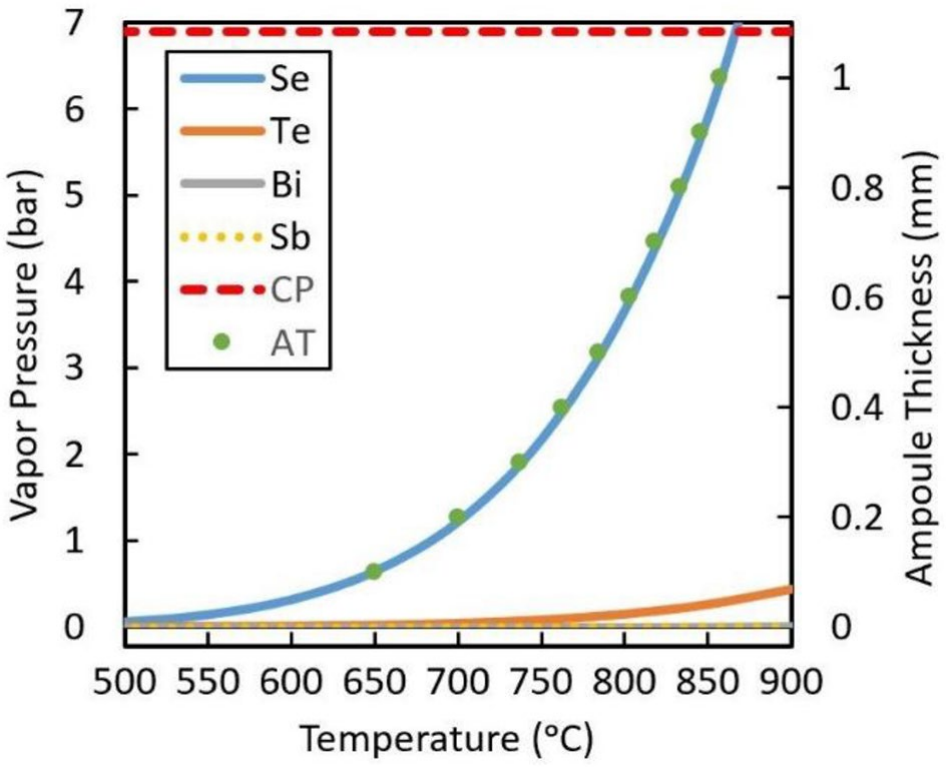
The Clausius–Clapeyron equation has been used to describe the relationship between temperature and vapor pressure for the BSTS system elements starting from 500 to 900 °C with ampoule thickness as a third axis. (CP) is the critical point at which the ampoule equation limits at, and (AT) is the ampoule thickness of the quartz tube.

The boiling point of Se is 685 °C; therefore, increasing the temperature up to 850 °C causes the partial pressure to increase inside the ampoule, making it a pressurized vessel and a fire hazard. Figure 1 presents theoretical data for BSTS elements and their partial pressure while varying the temperature described by Clausius–Clapeyron equation^27–29^. The Se partial pressure after 650 °C is very large, while the partial pressure of Se at 850 °C is 5.9 bar, which is equal to 85.57 psi and very close to the boundaries of the internal pressure ampoule formula with a value of 6.8 bar resembled by the critical point line in red. A potential solution is to have a thicker ampoule wall to sustain the pressure, but the pressure that the ampoule would withstand needs to be calculated to ensure it passes the safety requirement for the growth. Even so, a thicker ampoule still would be pressurized, meaning the hazard still exists, and upon failure, the results would be catastrophic. Thus, the main recommendation is to lower the processing temperature, preventing pressure from increasing inside the ampoule without losing efficiency or increasing growth time, but to date, no recommendations exist for what temperatures would satisfy the thermodynamic growth requirements for BSTS.

In this work, we set out to establish design principles for growing BSTS at low temperatures by leveraging thermodynamic modeling. We hypothesized that it is possible to grow high-quality BSTS single crystals at lower temperatures and that this modeling approach can be used to better understand how the elements interact with each other and form the compound(s) of interest. We show that growth conditions can be reduced from 850 °C to as low as 350 °C. Moreover, we show that this lower temperature processing route can provide better control over composition and therefore a higher quality BSTS single crystal under safer conditions because of reduced Se volatilization.

## Methods

### CALPHAD approach

Thermodynamic modeling was carried out by using CALPHAD software provided by ThermoCalc database “Alloy Solution database V6.0”. Pseudo-binary phase diagram was constructed for the BSTS system with a Bi–Sb–Te–Se fixed ratio of 1:1:1:2. Model conditions were constrained to temperatures between 0 and 650 °C at 1 atm. Although the BSTS composition exists at a single composition, we performed CALPHAD modeling over a compositional range 0–30% moles to produce more easily interpreted 2D phase diagram showing other phase fields that the process goes through.

### Thermodynamic modeling

The property model calculator predicts material properties based on their chemical composition and temperature. The calculator can compute the driving force, interfacial energy, liquids and solidus temperature, phases fraction, and phases activities, thus graphing the properties as contour plots or thermal maps^30,31^. For the BSTS alloy, the interest was to calculate Gibbs’ energy of formation plotted as a thermal map and amount of phase formation plotted as a contour plot. Any desired property would be calculated and shown as a third axis while the main calculation would be done as a function of temperature versus composition.

### BSTS elemental vapor pressure calculations

The Clausius–Clapeyron equation has been used to describe the relationship between temperature and vapor pressure for the BSTS system $\ln \left( {\frac{{P_{1} }}{{P_{2} }}} \right) = \frac{{ - \Delta H_{vap} }}{R}\left( {\frac{1}{{T_{1} }} - \frac{1}{{T_{2} }}} \right)$, where P_1_ is the known vapor pressure at a known temperature in atm, P_2_ is the vapor pressure of interest in atm, T_1_ is the corresponding temperature for P_1_ in K, T_2_ is the temperature of the point of interest in K, ΔH_vap_ is the enthalpy of vaporization in J/mol and R is the universal gas constant 8.314 J/(mol*K). The following is an example calculation of Se vapor pressure at 700 °C. The known temperature and pressure at the Se melting point are T_1_ = 494 K and P_1_ = 1.283e-5 atm, respectively. Converting the temperature of interest from Celsius to Kelvin yields T_2_ = 973 K. The heat of vaporization is ΔH_vap_ = 95,480 J/mol, and the gas constant is R = 8.314 J/(mol*K). Solving for P_2_, P_2_ = 1.2 atm = 1.22 bar. $\ln \left( {\frac{{1.283{\text{e}} - 5{ }}}{{P_{2} }}} \right) = \frac{ - 95480}{{8.314}}\left( {\frac{1}{494} - \frac{1}{973}} \right)$, solving for P_2_, P_2_ = 1.2 atm = 1.22 bar. In addition, the internal pressure of an ampoule formula is $S = \frac{P*r}{t}$, where S is hoop stress in Pa, P is working pressure in Pa, r is the inside radius of the ampoule in mm and t is ampoule wall thickness in mm.

### Solidification calculator Scheil simulation

Scheil simulations were used as a solidification calculator for estimating the solidification range of an alloy and the phases formed at various temperatures shown as a contour plot. This simulation provided a precise prediction of the diffusion in the solid state, solidification range of an alloy, phase formation, and composition in comparison to equilibrium solidification calculation. Additionally, it calculated the alloy segregation profile as a composition concentration gradient from the liquid phase to the solid phase using the equation $C_{s} = k*C_{0} \left( {1 - f_{s} } \right)^{k - 1}$, where C_s_ is the concentration of solute in the solid, C_0_ is the initial concentration of the liquid. f_s_ is the fraction solidified and k is the partition coefficient.

### Materials and synthesis

Reagent precursors metals Bi, Sb, Te, Se were ordered from Sigma-Aldrich Co., purity 5N grade. Quartz tubes were ordered from Technical Glass Products, Inc. with an outer diameter of 14 mm, inner diameter of 12 mm, and wall thickness of 2 mm for the ampoule. The raw materials were prepared with a Bi:Sb:Te:Se ratio of 1:1:1:2. The material total weight was 3 g mixed and crushed for 30 min, producing mixed fine powder. The powder was then loaded into the quartz tube to prepare for sealing. The inner tube wall was carbon-coated, preventing any possible reaction of the materials to the tube wall. Afterward, the tube was flushed with argon gas four times, displacing air, followed by vacuum pumping to reach a pressure below 10^−6^ torr to allow crystal growth in a vacuumed atmosphere. Finally, the tube was sealed by a hydrogen gas torch to a length of 6–8 cm.

### Sample characterization

After BSTS crystal growth, the ampoule was gently broken and the material was cleaved via razor blade and exfoliated to produce a thin layer for elemental composition detection using energy-dispersive X-ray spectroscopy (EDS, HITACHI S-4800 High Resolution Field Emission Scanning Electron Microscopy (SEM)) operating at an acceleration voltage of 20 to 30 kV. Both solid and powder samples were prepared for both crystal plane and powder X-ray diffraction (XRD, Philips PANalytical X’Pert, Cu $K_{\alpha }$ wavelength). For peak identification and crystal structure Rigaku data analysis software (PDXL—version 2) was used. Part of the crystal was prepared via fracturing for Thermo-gravimetric analysis (TGA, TA Instruments Discovery SDT 650).

## Results and discussion

### Hume–Rothery rules

BSTS is a quaternary alloy that has been analyzed based on the Hume–Rothery rules. Despite the difficulty increase in the multi-component system to analyze using simple binary phase diagrams, it is a good starting point to analyze the system using Hume–Rothery rules getting a few incites that can be confirmed by constructing a pseudo-binary phase diagram using an advanced phase modeling program such as CALPHAD approach ThermoCalc. Therefore, starting with the evaluation of all binary systems of the BSTS yielded 33.3%, meaning that the BSTS alloy results in a multiphase alloy that in terms of a solid solution, strengthening is unfavorable because multiphases tend to make the material brittle. However, the alloy was designed intentionally in a way to utilize the multiphase structure to create a semiconductor material that has a property of a conductor through the bulk and an insulator on the surface. Table 1 shows the elemental properties that are necessary for the evaluation starting with atomic size^32^. The atomic size difference criteria for the BSTS alloy is 50% in favor of solid-solution strengthening where the binary alloys of BSTS (Bi–Te, Bi–Sb, Te–Sb) have satisfied the condition where the atomic size difference must be less than 15%. The crystal structure criteria for the BSTS alloy is 33.3% in favor of solid-solution strengthening. The crystal structure of Bi is the same as Sb, indicating high solubility can occur, and the same is true for Te and Se. However, limited solubility occurs between Bi(Te,Se) binary alloys, and the same is true for Sb(Te,Se) binary alloys because of crystal structure differences. The valence criteria for the BSTS alloy is 33.3% in favor of solid-solution strengthening. The valence of the BSTS elements has the same conclusion as crystal structure criteria on predicting and evaluating solid-solution strengthening where Bi and Sb have the same valence and Te and Se have the same valence. However, Bi(Te,Se), Sb(Te,Se) binary alloys have different valences that limit solubility. Lastly, large differences in electronegativity cause intermetallic phase formation that limits the solubility between elements. The electronegativities of the BSTS elements are marginally different and dictate that solubility is limited, resulting in multiphase formation. Thus, Hume–Rothery rules predict that BSTS has poor solubility and leads to an alloy that contains multiphases. The possible phases that the binary phase diagrams suggested are hexagonal, rhombohedral, and trigonal. However, our system is quaternary and therefore we must construct a pseudo-binary phase diagram as opposed to a simple binary phase diagram.

**Table 1:** BSTS elements and their properties, where El is the elements, AR is the atomic radius, CR is the crystal structure, V is the valance, EN is the electronegativity, Rhom is the rhombohedral crystal structure, and Hex is the hexagonal crystal structure.

Hume–Rothery rules												
Physical properties					Atomic size difference %				Intermetallic compound formation			
El	AR (pm)	CS	V	EN	Bi	Te	Se	Sb	Bi	Te	Se	Sb
Bi	155	Rhom	3	1.9	0	7.7	25.2	6.5	Some binary phase diagrams have solubility limits			
Te	143	Hex	4	2.1	7.7	0	18.9	1.4				
Se	116	Hex	4	2.55	25.2	18.9	0	20				
Sb	145	Rhom	3	2.05	6.5	1.4	20	0				

### BSTS pseudo-binary phase diagram

**Figure 2:**
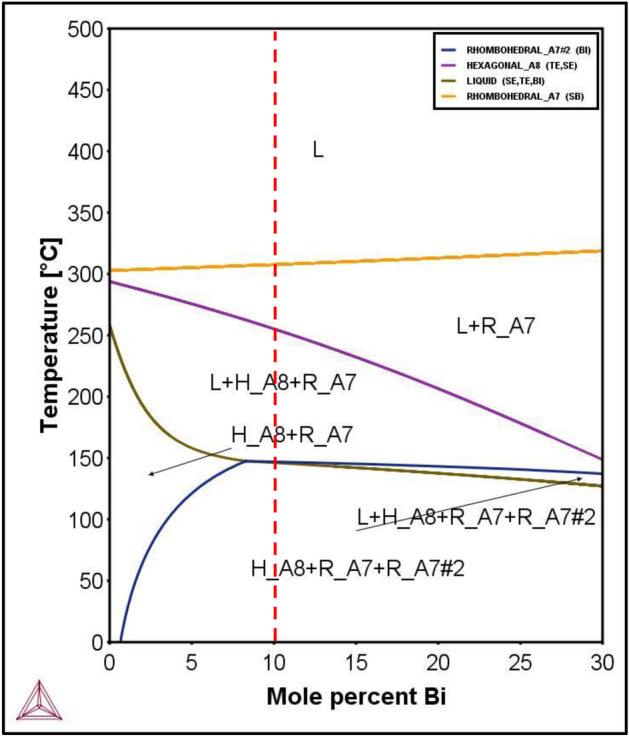
The pseudo-binary phase diagram of the BSTS alloy.

The analysis sequence started with constructing pseudo-binary phase diagram followed by thermal mapping of thermodynamic properties of the phases created. The pseudo-binary phase diagram can be analyzed to visually show the liquidus line that the crystal growth can start from. In addition, the graphs will indicate the stable phase at room temperature for the given composition of BSTS. Analysis of the pseudo-binary BSTS diagram shows a temperature of as low as 350 °C where a single-liquid phase exists. Therefore, single-crystal growth could be achieved thermodynamically at 350 °C as opposed to a higher temperature. The analysis of the pseudo-binary phase diagram suggests that the stable phases at room temperature are hexagonal and two rhombohedral phases. Figure 2 presents the pseudo-binary phase diagram of BSTS where the initial parameters of temperature, pressure, and composition were modeled by ThermoCalc software in accordance with the lab environment (P = 1 atm, T = 25 °C) where the red vertical line represents the composition with a Bi–Sb–Te–Se ratio of 1:1:1:2.

### Thermodynamic modeling

**Figure 3:**
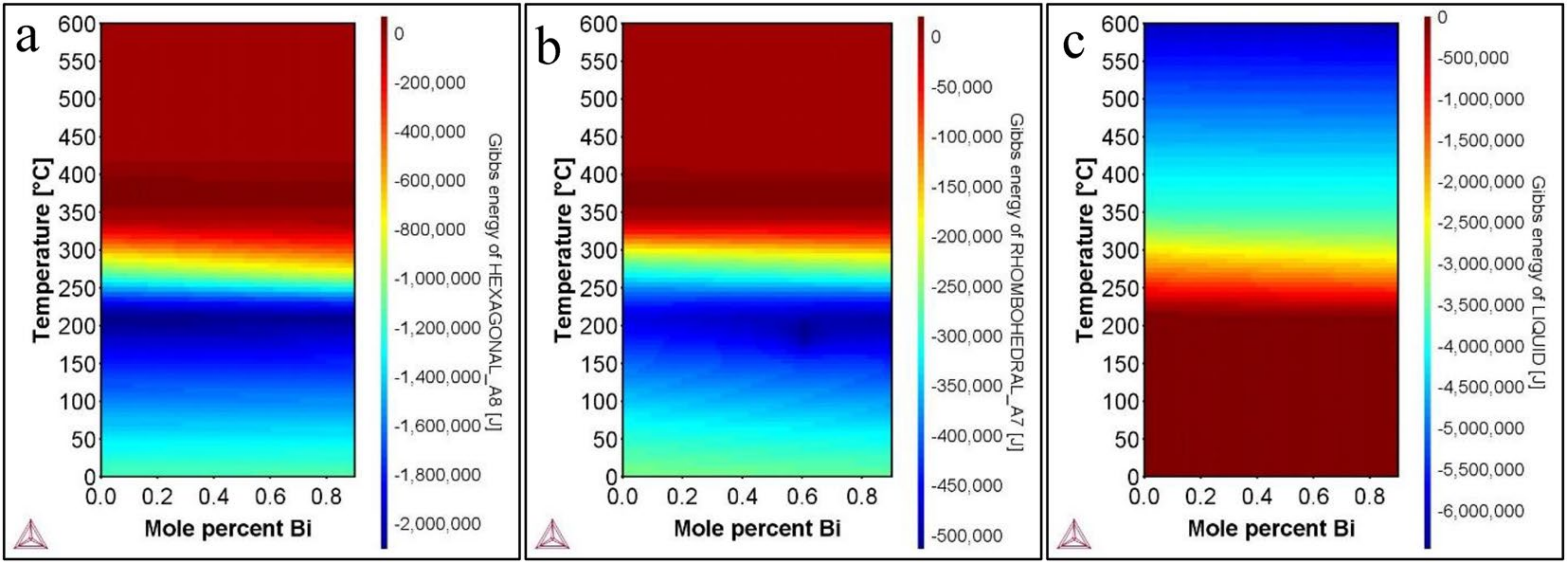
Thermal map of Gibbs free energy for the BSTS phases through various temperatures. (**a**) Gibbs free energy of hexagonal phase in the system. (**b**) Gibbs free energy of rhombohedral phase in the system. And (**c**) Gibbs free energy of liquid phase in the system.

Clearly, the pseudo-binary phase diagram suggests that from a thermodynamic standpoint, multiple phases should form when growing the BSTS ratio of 1:1:1:2. Nevertheless, previous reports including our own show that a single phase of BSTS can form in a single crystal with trigonal R-3m symmetry^33^. Therefore, it can be assumed that kinetics plays a substantial role in the formation of a phase over others. Indeed, our analysis of gibs free energy of different phases shows very different stabilities for the phases hexagonal, rhombohedral-1, and rhombohedral-2. Figure 3 shows the thermal map of Gibbs energy for the BSTS. For example, at 300 °C, the hexagonal phase Gibbs energy is about − 700 kJ, as shown in Fig. 3a, whereas the rhombohedral phase Gibbs free energy at the same temperature is about − 175 kJ, as shown in Fig. 3b. Therefore, the hexagonal phase is four times more likely to form than the rhombohedral phase and could explain the kinetics of these competing phases as well as the growth of the hexagonal phase in the final crystal, growth is dictated by the kinetics factor that ultimately explains the growth of phase over the over.

### Material phase properties and Scheil simulation

**Figure 4:**
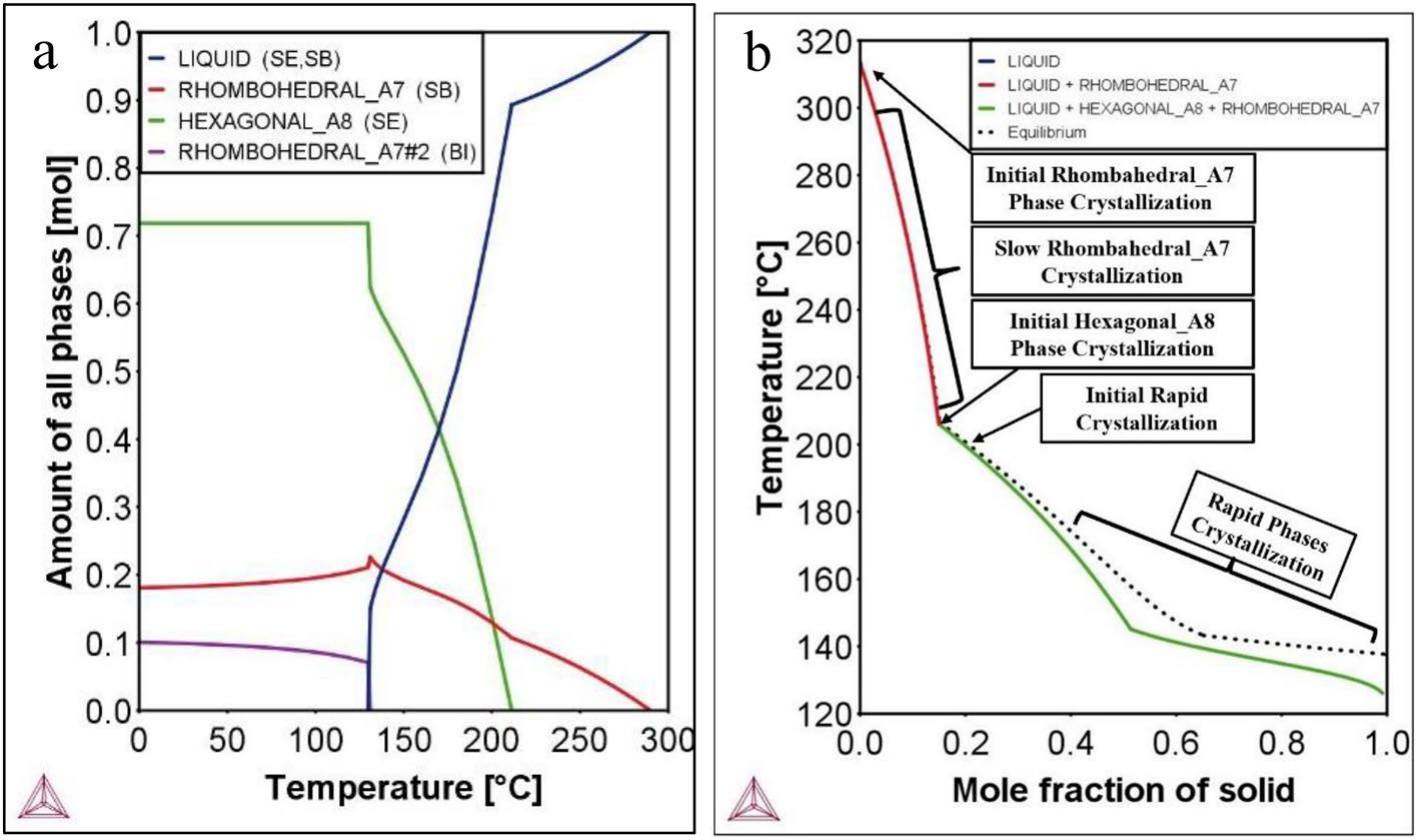
Material phase properties of BSTS. (**a**) Line plot of the phase concentrations versus temperature. (**b**) Scheil simulation.

The phase concentrations present at room temperature are found to be approximately around 70% hexagonal and 30% rhombohedral. It may perhaps be observed without straying too far afield from our primary focus that in experimental practice, this mixture of phases is avoided regularly. Very often, single-phase growth is achieved. In our previous studies., it has been ascertained that the single crystal grown has a rhombohedral crystal structure with space group ${\text{R}}\overline{3}{\text{m}}$^33,34^. Figure 4a shows the concentrations of phases created under various temperature ranges. Also, the graph shows exactly the starting/ending point of a phase. Notedly, it indicates that some liquid will form around 125 °C and that the liquid amount is approximately 20% around 150 °C. However, for single-crystal growth, the required starting amount of liquid needs to be 100%. In this study, Scheil simulation was used to show the critical point at which the phase starts to micro-segregate to form a solid phase in BSTS alloy^35,36^. Figure 4b shows interesting results. For example, starting at 320 °C, a full liquid BSTS forms a rhombohedral phase when cooled (red line), and during the cooling process, no sign of segregation is observed. At about 200 °C, a new phase forms and a small deviation occurs, separating the equilibrium line from the phase line where the micro-segregation starts (green line). The behavior of solidification changes around a 0.5-mol fraction nearly at 140 °C because the increase in hexagonal phase stability is roughly equal to six times the rhombohedral phase according to Gibbs free energy diagrams, where at higher temperatures it was around four times, as mentioned previously.

### Experimental validation

**Figure 5:**
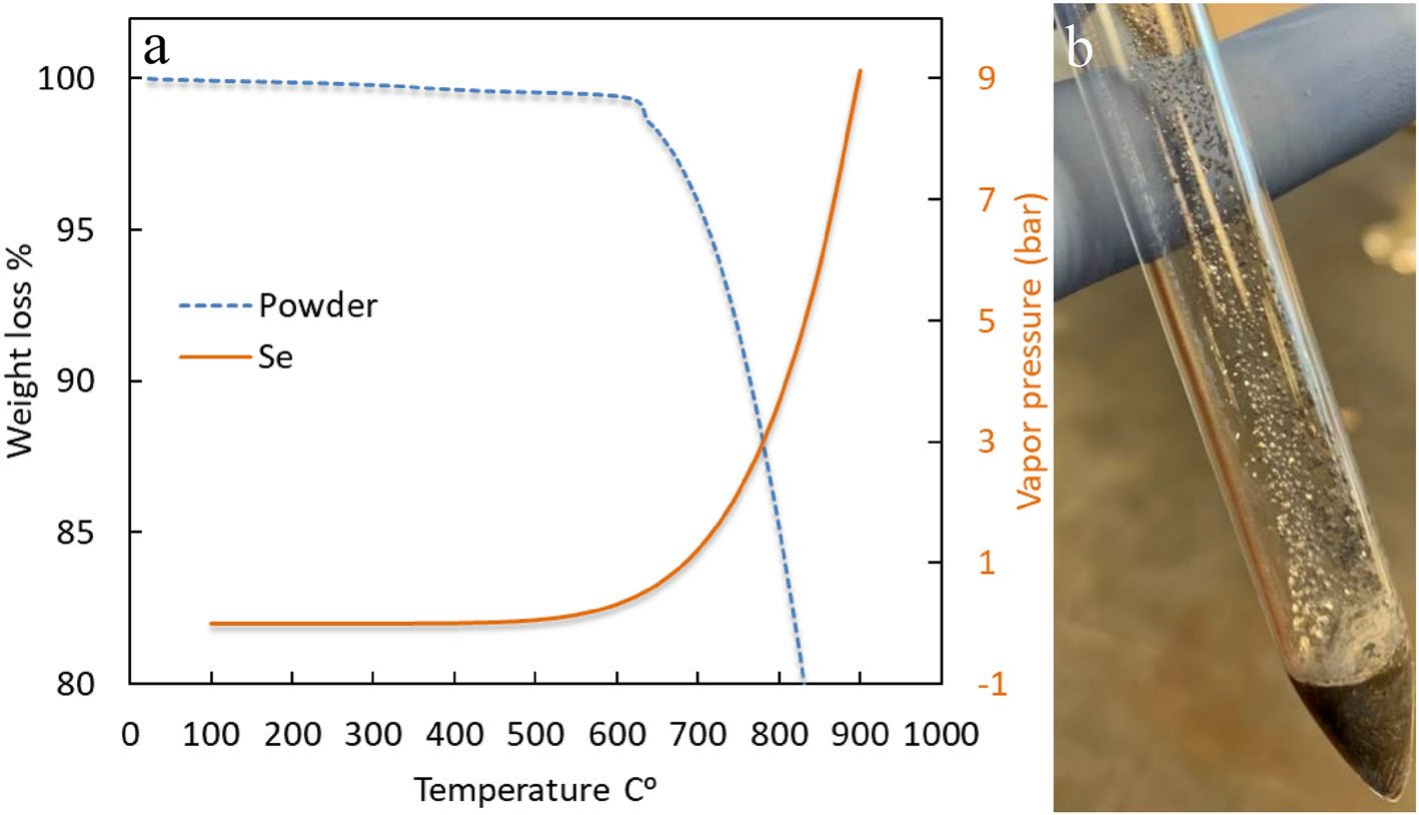
BSTS TGA of a synthesized sample. (**a**) BSTS TGA weight loss of material versus Se vapor pressure. (**b**) Synthesized BSTS sample.

A BSTS crystal has been synthesized following our previous work^34^, and the sample has been tested to validate the calculated result of ThermoCalc and to determine the optimal growth temperature. Figure 5a shows the BSTS thermogravimetric analysis (TGA). It is noticed that at around 630 °C, material loss commences. As a point of reference, the Se partial pressure calculated from Fig. 1 has been plotted at the same value in Fig. 5a. The material loss is explained by the increasing Se partial pressure. Therefore, the BSTS TGA results suggest that the growth temperature critical point is 630 °C. Our recommendation to achieve higher quality crystals is to grow BSTS while maintaining a temperature lower than 630 °C. Figure 5b illustrates a Bridgman crystal growth of BSTS, setting the three zones as 670–770–500 °C. The results show that some material evaporated from the bulk sample deposited on the ampoule wall because of the elevated temperature passing the threshold.

After growing the BSTS crystal and running the TGA test on, we decided to validate the ThermoCalc graph further and test out several temperatures for crystal growth. These three samples were grown using melt growth method. The chosen temperatures are 350 °C, 450 °C, and 550 °C. Parameters of growth were carried out identically with one change only, holding temperature. The temperature increases by an increment of 100 °C synthesizing in total three samples. A good sample was defined by three main characteristics: homogeneity, chemical composition accuracy and cleavability. Homogenous crystals were identified by showing even elemental distribution throughout the crystal by examining multiple areas of the crystal. Chemical composition accuracy is the most challenging aspect for all types of crystals and therefore it became the focus of this study in particular. Cleavability, in this study, is the qualitative ease with which a thin layer of BSTS crystal can be mechanically exfoliated.

**Figure 6:**
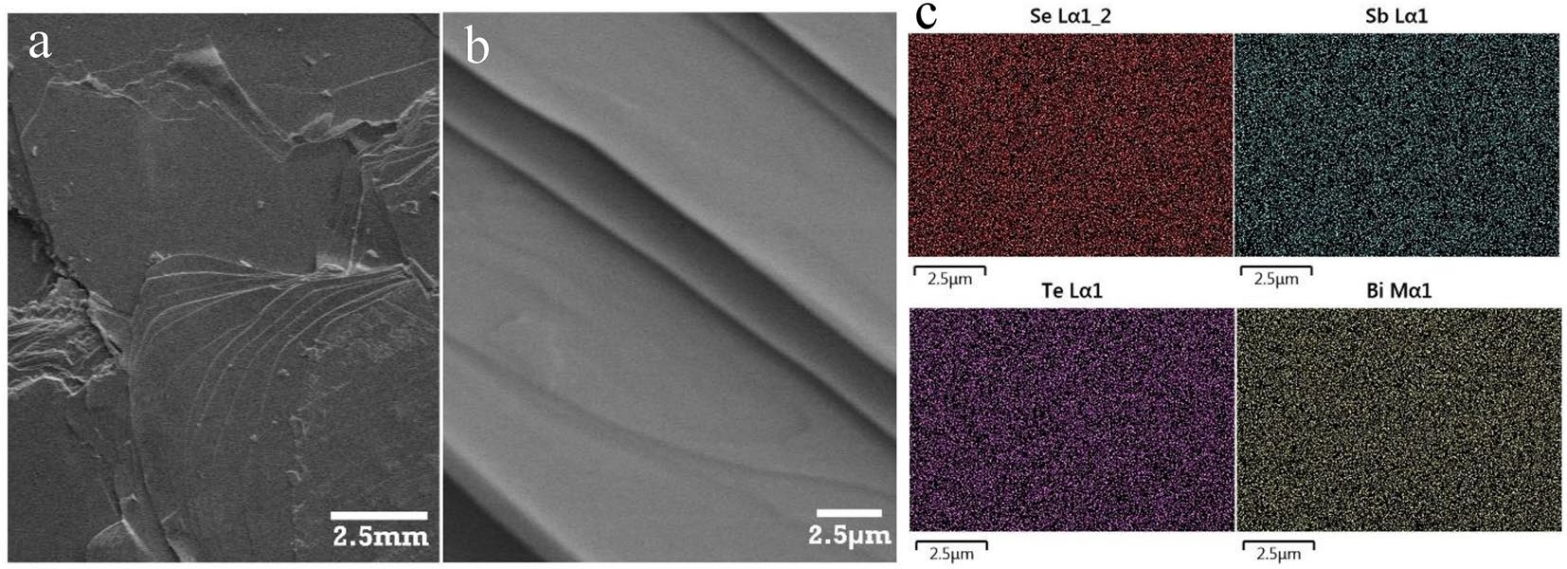
BSTS SEM and EDS. (**a**) BSTS low magnification SEM. (**b**) BSTS high magnification showing the crystal layers. (**c**) BSTS EDS showing a homogenous elemental distribution.

EDS results are summarized in Table 2. While slight differences in composition are reported, none of these are statistically meaningful differences given the large error (> 5wt%) common to EDS. However, the sample fabricated at the lowest temperature of synthesis. 350 °C, was notably more challenging to cleave. At the same time, increasing synthesis temperature tended to improve a crystal’s cleavability. Figure 6a–c is BSTS SEM showing single crystal layers and an EDS showing crystal’s elemental distribution homogeneity.

**Table 2:** EDS results at different growth temperatures for BSTS crystals.

Element	Bi	Sb	Te	Se	Cleavability
Mole ratio	1.00	1.00	1.00	2.00	–
350 °C	1.01	1.00	1.01	1.96	Difficult
450 °C	0.96	1.09	1.00	1.95	Hard
550 °C	1.05	0.95	1.02	1.90	Easy

**Figure 7:**
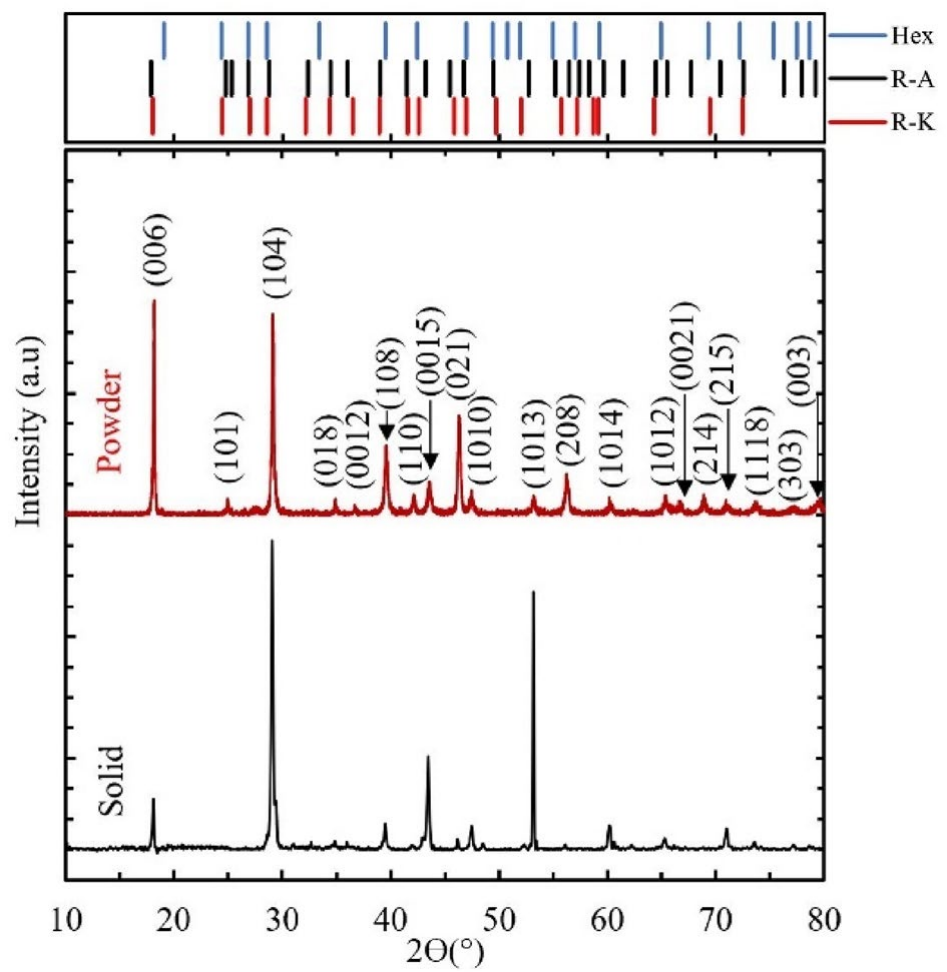
BSTS crystal XRD pattern of solid and powder samples. Where (Hex) is Telluronevskite (Bi_3_Se_2_Te) hexagonal structure, (R–A) is antimony selenium telluride (Sb_2_SeTe_2_) rhombohedral structure and (R–K) is Kawazuite (Bi_2_Te_2_Se) rhombohedral structure.

Part of the grown BSTS single crystal was used for phase identification using XRD for both powder and solid patterns Fig. 7. The pattern shown is from a BSTS sample grown at 450 °C. The diffraction peaks correspond to a single-phase crystal in either the hexagonal or rhombohedral space group. A complete disambiguation of which phase will be the focus of future work.

## Conclusion

Hume–Rothery rules can predict solid-solution homogeneity formation accurately for binary alloys. Any elements can be used as substitutional elements or added to the mix once they follow the rules to predict their behavior. As for complex alloys, more complex methods such as the CALPHAD approach are needed to empower prediction accuracy. CALPHAD approach has been used to determine the optimum conditions theoretically for BSTS single-crystal growth and has given valuable insights about dominant phases and their quantity. Scheil simulation provides an overview of the BSTS solidification process. TGA has confirmed that the critical point of crystal growth temperature is 630 °C, at which point material starts to evaporate from the bulk sample. BSTS EDS and XRD confirms the possibility of crystal growth at low temperatures resulting in better crystal quality in terms of chemical composition accuracy.

## Data Availability

Raw data were generated at University of Utah. Derived data supporting the findings of these study are available on request.
